# Clinical Characteristics, Comorbidities, and Treatment in Patients with Pemphigus—A Single-Center Retrospective Study

**DOI:** 10.3390/antib13040103

**Published:** 2024-12-13

**Authors:** Natalia Welc, Sandra Ważniewicz, Paweł Głuszak, Maciej Spałek, Agnieszka Seraszek-Jaros, Magdalena Jałowska, Marian Dmochowski

**Affiliations:** 1 Autoimmune Blistering Dermatoses Section, Department of Dermatology, Poznan University of Medical Sciences, 61701 Poznan, Poland; sandrawazniewicz@gmail.com (S.W.); paw.gluszak@gmail.com (P.G.); mspalek@ump.edu.pl (M.S.); mjalowska@ump.edu.pl (M.J.); mdmochowski@ump.edu.pl (M.D.); 2 Department of Bioinformatics and Computational Biology, Poznan University of Medical Sciences, 61701 Poznan, Poland; seraszek@ump.edu.pl

**Keywords:** pemphigus, comorbidity, treatment, epidemiology

## Abstract

Background/Objectives: Pemphigus comprises a diverse group of disorders within the autoimmune bullous dermatoses (AIBDs) spectrum. Among these, pemphigus vulgaris (PV) and pemphigus foliaceus (PF) are the most commonly encountered variants. Despite its rarity, this condition can pose a life-threatening risk. We aimed to assess clinical characteristics, comorbidities, medication, as well as the treatment of various types of pemphigus in pemphigus patients. Methods: We gathered data from 69 patients treated in the Department of Dermatology in the years 2016–2023. The investigation included sex, age at diagnosis, type of pemphigus, comorbidities and medications, presence of neoplasms and treatment of pemphigus, as well as enzyme-linked immunosorbent assay (ELISA) and direct immunofluorescence (DIF) results. The data were statistically analyzed with the *p*-value set at 0.05. Results: The study group comprised 69 patients, including 41 women and 28 men. The mean age at diagnosis was 56.89 years +/− 15.42 years. A total of 79.31% of the patients were diagnosed with PV and the following 26.09% with PF. The most common comorbidities were arterial hypertension, hypercholesterolemia, and diabetes mellitus. The dominant treatment regimen was the systemic use of glucocorticosteroids (GCSs; 90% and 94% of PV and PF patients, respectively). More than half of the patients received at least one GCS-sparing treatment, including dapsone and rituximab. We observed a significantly frequent presence of IgG deposits in DIF in patients with PF (*p* = 0.0217) and a subsequent correlation between the concurrent presence of IgG deposits in DIF and anti-DSG1 antibodies in ELISA testing (*p* = 0.0469). The combination of IgG, IgG1, IgG4, and C3 deposits was more often existent in PF patients (*p* = 0.0054) and the combination of IgG4 and C3 deposits in PV patients (*p* = 0.0339). We also found a positive correlation between the level of anti-DSG1 antibodies and the age at diagnosis (*p* = 0.0298). Conclusions: Patients with pemphigus are very often diagnosed with significant comorbidities and take diverse medication, which shows that the treatment of pemphigus should follow a multidisciplinary approach. Accurate analysis of the clinical condition of the patients, as well as the results of the ELISA panel or DIF, is crucial for a successful diagnostic and therapeutic process.

## 1. Introduction

Pemphigus is a family of heterogeneous diseases from the spectrum of AIBDs, which includes pemphigus vulgaris (PV) and pemphigus foliaceus (PF) as the most frequently observed types of pemphigus. The disease, though relatively rare, can be life-threatening [1].

The mechanism of pemphigus lies in the immune response targeting desmoglein 3 (DSG3) and, to a lesser extent, desmoglein 1 (DSG1) [2]. The diagnosis relies on examining the perilesional tissue through direct immunofluorescence (DIF), conducting a series of biochemical and molecular tests, and, most importantly, thoroughly analyzing the clinical presentation of patients to guide appropriate laboratory testing selection [3].

The range of symptoms and the disease’s course can greatly differ among patients, but it consistently tends to affect natural body orifices [4,5]. In these areas, acantholysis leads to the formation of blisters located above the basal layer, typically filled with clear fluid. These blisters rupture easily, resulting in erosions that usually heal without scarring. Many cases also exhibit changes in the mucous membranes of the oral cavity, especially the palate. Skin appendages like hair follicles, eccrine glands, and nail structures may also be affected, given that DSG1 and DSG3 are expressed there [6].

Epidemiological aspects of pemphigus vary considerably in different global regions. Over the years, there have been significant shifts in the prognosis, mortality rates, and clinical outcomes for pemphigus patients. Additionally, due to limited and inconsistent observational studies, a clear understanding of comorbidities and associated conditions in pemphigus patients still needs to be discovered.

Our study aimed to provide an account of the epidemiology, treatment, and comorbidities associated with various subtypes of pemphigus observed in patients diagnosed with pemphigus in the Greater Poland region over seven years.

## 2. Materials and Methods

Data concerning patients diagnosed with pemphigus in the Department of Dermatology from 2016 to 2023 were collected. These data included parameters such as sex, age at diagnosis, type of pemphigus, comorbidities and medications taken, presence of neoplasms, and treatment of pemphigus. If available, the levels of anti-DSG1 and anti-DSG3 antibodies and the result of DIF were also considered. Statistical analysis was performed, and the *p*-value below 0.05 was set as significant.

## 3. Results

### 3.1. Epidemiology

Through 2016–2023, 69 patients were diagnosed and treated with pemphigus in the Department of Dermatology, of whom 59.42% (*n* = 41) were female and 40.58% (*n* = 28) were male. The female-to-male prevalence ratio of PV was calculated as 3:2, whereas in PF it was 5:4. Out of 69 patients, 51 were diagnosed with PV (73.91%) and 18 with PF (26.09%). In the group of PV, in 10 patients, the mucosal dominant form of PV was observed. In three cases, drug-induced pemphigus was suspected: a 29-year-old male treated for depression with escitalopram, a 67-year-old male treated for arterial hypertension with indapamide, and an 83-year-old female treated for arterial hypertension with ramipril. The mean age at the diagnosis of the study group was 56.89 years +/− 15.42 years, which for the patients with PV and PF were, respectively, 55.22 years +/− 14.38 years and 61.56 years +/− 17.64 years.

### 3.2. Comorbidities and Neoplasms

The most frequent comorbidities observed in the study group, both in PV and PF patients, belong to cardiovascular and metabolic diseases, such as arterial hypertension (41% and 50% of the patients, respectively), hypercholesterolemia (27% and 17%), diabetes mellitus (20% and 11%), coronary artery disease, and/or cardiac failure, including cardiac infarction (14% and 17%). Several patients reported osteoporosis, hyper- or hypothyroidism, and psychiatric, renal, pulmonary, or gastrointestinal disorders. However, no significant difference was found between the two subgroups. A full list of the comorbidities is presented in Table 1.

Out of 69 pemphigus patients, 10 patients in total, which stands for 14% of patients with PV and 17% of patients with PF, were diagnosed with any neoplasm in their life. No significant difference was observed between these groups. Table 2. comprises all neoplasms found in the patients’ medical history.

### 3.3. Diagnostics and Treatment

The result of the ELISA assessment of the levels of antibodies to DSG1 and DSG3 was available for 65 out of 69 patients (94.2%). The value of the anti-DSG-1 level in patients with PF was significantly higher than in patients with PV (*p* = 0.0031). Table 3 displays a detailed analysis of the ELISA test for the study group. Moreover, we found a slight positive correlation between the level of anti-DSG1 antibodies and the age at diagnosis (*p* = 0.0298). The analysis is presented in Table 4.

During the diagnostic process, the DIF was used in 67 out of 69 patients (97.1%). The method detected IgG, IgG1, IgG4, C3, and IgA deposits. In 28.99% of patients, only one type of deposit was discovered, and several combinations were noted in the rest of the study group. The most prevalent one was the coexistence of IgG4 and C3 deposits (17 cases). We found a significantly frequent presence of IgG deposits in patients with PF (*p* = 0.0217). Moreover, a correlation was noticed between the concurrent presence of IgG deposits in DIF and anti-DSG1 antibodies in ELISA testing (*p* = 0.0469). We also observed that the combination of IgG, IgG1, IgG4, and C3 deposits was more often existent in PF patients (*p* = 0.0054) as well as the combination of IgG4 and C3 deposits in PV patients (*p* = 0.0339). All data, including the results from DIF tests, are summarized in Table 5, Table 6 and Table 7.

Various treatment choices were made according to the clinical condition of the skin and mucosa, comorbidities, and previous treatment failure. GCSs, as the first-line treatment for pemphigus, were the most frequently used drugs in both PV and PF patients (90% and 94% for systemic GCSs, 63% and 83% for topical GCSs, respectively). In some individuals, topical calcineurin inhibitors were necessary (8% of patients with PV, 17% of patients with PF).

Given that a considerable fraction of the study group had cardiovascular or metabolic comorbidities, GCS-sparing treatment, including dapsone (DP), rituximab (RTX), intravenous immunoglobulins, or cyclophosphamide, was introduced. More than half of the patients required any of the mentioned medications, and some of them had to be treated with two or more of these pharmaceuticals (52.17% and 11.59%, respectively). GCS-sparing treatment was vital, especially in patients with PV, of whom 54.9% received at least one GCS-sparing drug. DP and RTX were the most frequent choices; DP was administered in twenty-three cases of PV (45%) and seven patients with PF (39%), and RTX in eight instances of PV (16%) and four patients with PF (22%). Detailed data are enclosed in Table 8.

## 4. Discussion

### 4.1. Epidemiology

In the group of patients included in this study, PV predominates, and in both phenotypes, the disease affects women more often, as reflected in data from the literature [7]. The ratio of PV to PF in this study is 3:1, consistent with the literature, which generally reports a prevalence ratio of approximately 3:2 for PV and PF in European populations. However, several cross-sectional studies from European countries—Spain, Turkey, Romania, and Israel—have reported higher PV-to-PF ratios, likely attributable to endemic variations and genetic predispositions within those populations [8,9,10,11,12]. Outside Europe, PF is more frequent in Tunisia and Brazil, which is attributed to genetic susceptibility with endemic subtypes of the disease identified [12,13,14].

While European studies suggest a slightly higher prevalence of pemphigus in females compared to males, similarly to our results, global epidemiological data suggest no significant sex-based differences in pemphigus incidence [10]. Notably, only a Spanish study reported a female-to-male ratio of 1:1.2, probably due to the small sample size [15]. Other countries with male predominance in pemphigus diagnosis are Middle Eastern countries, such as Saudi Arabia or Kuwait [10,16]. Another similar study from Poland, conducted in a neighboring region, identified a female-to-male ratio of 2.88, markedly higher than in our cohort, despite the comparable ethnic background [17]. This discrepancy may reflect regional differences in genetic susceptibility, environmental factors, or methodological variations.

According to the literature, the mean age of pemphigus diagnosis in Europe is between 50 and 60 years old, which is consistent with our observations. The average age of diagnosis in our cohort for PV was about 55 years, and for PF, about 62 years. Similar results were obtained in a French study of 249 patients, 222 of whom had PV or PF, in whom the mean age of diagnosis was about 58 years for PV and about 61 years for PF [18].

### 4.2. Antibodies

In our study, we observed a significantly higher value of anti-DSG1 antibodies in patients with PF than in patients with PV, which could underscore the importance of these antibodies in PF pathogenesis, as PF patients mainly present antibodies against DSG1, in comparison to PV patients, who exhibit additional antibodies against DSG3 [19,20]. While analyzing the DIF results, we found that IgG deposits were significantly more frequently present in patients with PF (*p* = 0.0217), who tend to display anti-DSG1 antibodies more often. In our study group, we confirmed that the patients with IgG deposits more often displayed anti-DSG1 antibodies (*p* = 0.0469), which agrees with the literature. According to Jalowska et al., the difference in the structure of the cytoplasmic regions of the desmogleins (DSG1 has five repeat unit domains, whereas DSG3 has two) might be the reason for the more efficient binding of IgG by anti-DSG1 than by anti-DSG3 antibodies [21].

Furthermore, Weiss et al. noted that the levels of antibodies anti-DSG1 and anti-DSG3 might reflect the clinical disease severity [22]. As we did not monitor the levels of desmogleins over time, we were not able to compare our data in this matter. However, we discovered a slightly positive correlation between the age at diagnosis and the level of anti-DSG1 antibodies (*p* = 0.0298), which was not yet acknowledged in any study and requires further investigation.

### 4.3. Drug-Induced Pemphigus

In our cohort, we identified three patients with suspected cases of drug-induced pemphigus. One case was associated with escitalopram, a selective serotonin reuptake inhibitor (SSRI) primarily used in the treatment of mood disorders, while the other two were hypotensive medications, indapamide and ramipril. The existing literature recognizes these medications as potential triggers for various AIBDs, particularly bullous pemphigoid [23,24]. However, exposure to thiol drugs, such as captopril or penicillamine, was noted in the literature [25]. Thus, we emphasize the importance of careful evaluation of a patient’s medication history, especially the recent introduction of new drugs to exclude the possibility of drug-induced AIBDs.

### 4.4. Comorbidities

**Figure 1:**
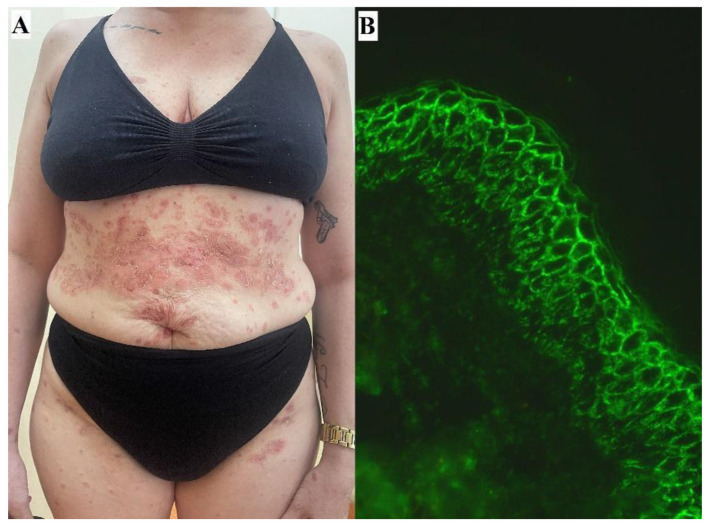
A representative female patient with PF in whom multiplex ELISA (Euroimmun, Lübeck, Germany) revealed grossly elevated levels of IgG antibodies to DSG1—11.17, whereas IgG antibodies to DSG3 were within a normal range (DSG3—0.22) (both cut-off values 1.0). The patient was successfully treated with systemic and topical GCSs and RTX. Shallow erosions covered with brownish crusts on the trunk, including the periumbilical area and permanent tattoos preceding PF lesions, can be seen (**A**). IgG (++), IgG1 (++), IgG4 (+++) (**B**), and C3 (++) pemphigus deposits were detected in DIF of perilesional skin and visualized with blue light-emitting diode technology-operated microscopy (original objective magnification ×40).

Regarding the PF patient with tattoos preceding the appearance of PF lesions (Figure 1), there are no data on triggering pemphigus diseases by tattooing. Nevertheless, immune responses that are not well-characterized may occur after tattooing [26]. Therefore, the relationship between the smoldering influences of tattooing and PF triggering cannot be ruled out in this case.

Arterial hypertension and type 2 diabetes were the most prevalent comorbidities among our patients; however, they preceded the diagnosis of pemphigus. Thus, we do not consider them an effect of GCS therapy. In such patients, GCS-sparing treatment should be regarded to avoid the progression of these comorbidities.

No significant association was found among our patients between the presence of PF or PV and other diseases, including cancer or cardiovascular diseases (CVDs), which may be attributable to the limited sample size.

The study by Pathak et al. showed a higher incidence of cardiovascular disease and psychiatric disorders, including anxiety, depression, insomnia, and chronic kidney disease, among others, in patients with AIBDs [27]. The authors indicate that the high prevalence of hypertension, diabetes mellitus, arthritis, and hyperlipidemia may be due to prolonged corticosteroid therapy. Heelan et al. reported an increased risk of hypothyroidism, diabetes, and inflammatory bowel disease (IBD) in patients with pemphigus. Moreover, the authors highlighted the importance of glucose testing at the initiation of corticosteroid therapy and ongoing monitoring during treatment for the early detection of hyperglycemia [28]. In many studies, it has been shown that patients with AIBDs have an increased risk of atherosclerotic cardiovascular disease, heart failure, arrhythmia, VTE, and CVD-related mortality [29]. Furthermore, the available literature on comorbidities in pemphigus suggests that anxiety and depression are also recognized risk factors for pemphigus recurrence, as they are associated with immunological dysfunction [30]. Female patients with pemphigus have been observed to have an increased risk of developing depression [31]. However, it is not known whether it is due to ethnic differences or attributable to the fact that females are more likely to be affected by the disease. In our cohort, no significant increase in mood disorders has been identified, probably due to a limited follow-up period and lack of systematic psychiatric screening.

The literature suggests that the possibility of PV and HIV co-occurrence should also be considered, as such cases are very rare but require special care. Due to the interaction of the two diseases with different immune-mediated mechanisms, an appropriately selected treatment is necessary, being aware of the serious adverse effects of immunosuppressive drugs [32].

### 4.5. Malignancies

In our cohort, the most frequently diagnosed malignancies were thyroid cancer (in two patients with PV) and breast cancer (one patient in the PV and one patient in the PF group). However, due to this study’s cross-sectional nature, we could not conduct longitudinal follow-up to determine if additional malignancies developed over time. This limitation is notable, as both PF and PV often precede the diagnosis of malignancy, suggesting a potential for further cancer development in these patients.

In a study by Schulze et al., 3.9% of patients with PV developed hematologic malignancies, 0.9% developed oropharyngeal carcinomas, and 3.7% were diagnosed with colon cancer. The authors noted that only 20% of PV patients had malignancies preceding the onset of this autoimmune disease, suggesting PV may act as a risk factor for the development of these malignancies. No increased incidence of oropharyngeal or gastrointestinal carcinomas was observed in patients with PF, potentially due to the lack of mucosal involvement in this form. However, 16.5% of PF patients were diagnosed with non-melanoma skin cancer, with the majority of cases occurring before the onset of AIBD, implying that the skin lesions characteristic of PF are unlikely to be a direct trigger for malignancy [33]. Kridin et al., on the other hand, indicate an increased risk of esophageal and laryngeal cancer in patients with PV, while their study did not confirm an increased incidence of gastrointestinal or colon cancer [34].

### 4.6. Mental Health

AIBDs significantly affect patients’ quality of life and have a significant impact on their emotional state and mental health [30]. Studies suggest that patients with pemphigus are more likely to suffer from anxiety and depressive disorders compared to the general population and patients with other chronic dermatoses. In addition, long-term use of GCSs, which are first-line treatment, is a risk factor for comorbid mental illness in pemphigus patients [35].

Kalinska-Bienias et al. used specific Autoimmune Bullous Disease Quality of Life (ABQOL) and the Treatment Autoimmune Bullous Disease Quality of Life (TABQOL) questionnaires to assess the quality of life in patients with AIBDs. Their study found that patients younger than 70 had a lower QOL score in the ABQOL questionnaire than in patients whose disease developed after 70. The authors emphasize the significant impact of itching, difficulty in wound healing, and depression on the quality of life of patients with AIBDs. In addition, lower questionnaire scores were noted in patients with PV than in those with BP, which may be due to less frequent mucosal involvement in the case of BP and less severe adverse effects due to response to lower treatment doses in patients with BP [36].

Studies suggest that the predisposing factor for the occurrence of depression in patients with PV is the female gender. The duration of the active phase of the disease and hospitalization have also been shown to be risk factors for the onset of psychiatric disorders. The authors underline the role of RTX, which, by having a rapid and prolonged action, allows for the improvement of the well-being of patients with PV [37].

### 4.7. Treatment

In many cases, the treatment of choice for patients with non-enzymatic autoimmune blistering diseases (n-eAIBDs) remains GCS therapy, both general and topical; however, due to their side effects, such as the development of diabetes mellitus, hypertension, osteoporosis, and gastrointestinal ulcers, there is a preference for GCS-sparing treatment. GCS-sparing treatment includes methotrexate (MTX), RTX, cyclosporine, azathioprine (AZA), mycophenolate mofetil (MMF), cyclophosphamide, or DP [38].

In our cohort, 16% of patients with PV and 22% with PF received RTX treatment at a dose of 1 g intravenously. A separate study conducted at our clinic demonstrated a significant reduction in GCS use and a decrease in pemphigus activity following RTX therapy. Notably, 81.82% of patients with severe AIBDs achieved partial or complete remission within one year of initiating RTX treatment [39]. In a case-control study, patients treated with RTX showed greater clinical and laboratory improvements compared to those receiving MMF or AZA. The authors point to fewer adverse events with the treatment with RTX [40]. RTX has demonstrated efficacy in patients with severe, recurrent PV and newly diagnosed pemphigus cases. Studies have shown that combination therapy with RTX and short-term prednisone is superior to GCS therapy alone. This approach facilitates a reduction in steroid dosage, minimizes associated side effects, and leads to complete remission in a more significant proportion of patients following prednisone withdrawal [41].

Patients included in our study were often administered additional GCS-sparing agents. Specifically, 45% and 39% of the PV and PF patients, respectively, were administered DP. DP, as an antimicrobial and anti-inflammatory drug, functions by inhibiting neutrophilic recruitment. Although it is primarily used as a first-line treatment in dermatitis herpetiformis and linear IgA bullous dermatosis, it is often utilized in various dermatoses, such as AIBDs. A retrospective study conducted in our clinic demonstrated that DP is effective in managing n-eAIBDs, as it facilitates prednisone dose reduction and can be safely and effectively used in this setting, particularly in milder cases [38].

Intravenous immunoglobulins (IVIGs) were administered to 8% of PV patients and none of the PF patients. IVIGs are typically reserved for very severe cases or for patients with n-eAIBDs who experience relapse following RTX therapy. When combined with RTX or other GCS-sparing agents, IVIGs are safe and effective, facilitating rapid symptom control and achieving remission [42]. In addition, RTX + IVIG combination therapy allows for lower doses of RTX and longer IVIG intervals [43]. Multidrug regimens have been shown to achieve a lower relapse rate compared to a prednisone/RTX regimen. It is recommended to add IVIG to the prednisone/RTX protocol, which eliminates pathogenic autoantibodies. It emphasized the importance of maintaining full clinical remission until the critical mass of autoreactive plasma cells disappears [44].

### 4.8. Mortality

Numerous studies show that mortality rates are significantly higher in patients with a diagnosis of pemphigus over 65 years of age. Kridin et al. report the most common causes of death in patients with pemphigus as infectious diseases, especially pneumonia, septicemia, and skin infections. A significant role is also attributed to cardiovascular diseases, malignancies, and cerebrovascular diseases [45]. In contrast, in the Taiwan study, the main cause of death was pemphigus itself [46]. One study conducted in France revealed the most common causes of death as malignancy, cardiovascular disease, infection, and dementia [18]. It has been shown that mortality due to infection is related to immunosuppressive treatment and glucocorticosteroid therapy. However, this risk is dose-dependent, and no increased risk has been observed with prednisone doses < 10 mg and cumulative doses < 700 mg [47]. This emphasizes the need for GCS-sparing treatment. In our cohort, 54.9% received at least one GCS-sparing drug, including DP and RTX. Fortunately, in our study group that comprised patients diagnosed with pemphigus in the years 2016–2023, no case of death has been reported yet, probably due to advanced diagnostics, simple access to DIF, disease-controlling treatment, and available advanced treatment methods, including RTX.

### 4.9. Genetic Aspects of Pemphigus and Potential Biomarkers

The specific gene, which could be the cause of pemphigus, is yet to be discovered. For now, the strongest genetic associations considering pemphigus were linked to human leukocyte antigen (HLA) class II alleles, especially in particular ethnic groups [25,48,49]. For instance, HLA-DRB1*0402 and DQB1*0302 alleles were discovered more frequently in the Jewish population [50], and the HLA-DQB1*0503 allele was associated with PV in non-Jewish populations [51]. However, such alleles as DQB1*0503 and DRB1*0402 seem to be present in both European, as well as North and South American populations [52,53,54,55]. The DRB1*03 allele was also described as the main susceptibility allele in Tunisian cases of PF [56]. Some single-nucleotide polymorphisms (SNPs) in different genes, such as TNF-alpha, IL-6, IL-10, CTLA4, ICOS, CD86, or ST18, which are a part of apoptosis or immune response, seem like potential markers of genetic liability to PV [57,58,59]. Mahmoudi et al. presented that knowledge of SNPs of glucocorticoid receptor genes NR3C1 and NUDT15, which take part in drug metabolism and pharmacokinetic and pharmacodynamic processes, could also lead to a more personalized approach to pemphigus treatment [60]. Studies suggest that increased lymphocyte Th2 and Th17 activity and reduced Treg activity might contribute to PV pathogenesis. Increased levels of IL-4, IL-6, and IL-17A and reduction in Treg cells and FOXP3 could follow as potential biomarkers of developing PV [61]. Moreover, high levels of nuclear c-Myc in perilesional keratinocytes were shown to be a promising marker for the diagnosis of early-stage PV [62].

There are also some novelties in terms of severity markers for pemphigus. Sar-Pomian et al. correlated the scalp involvement in pemphigus with generally higher disease severity, a longer time to complete remission, as well as its shorter duration [63]. Additionally, eye involvement could stand as an example of high disease severity that would require intensive immunosuppressive treatment, e.g., with RTX [64]. Zeid et al. recommended measurements of sPD1 in PV patients, as lower levels of sPD1 might follow a defective PD1 pathway and, eventually, serve as a severity marker for PV [65]. Interestingly, it was also found that MPV (mean platelet volume) is significantly lower in PV patients, especially in those with laryngeal involvement. As MPV is a part of a simple complete blood count, it could be used as a cheap and simple marker predicting this particular subtype of PV [66]. Nevertheless, there is still much more to be discovered in the subject of markers for the detection and severity of pemphigus diseases. As we could not collect data on our cohort’s genetic characteristics, we recommend that future studies implement these aspects in diagnosing and treating pemphigus patients.

### 4.10. Limitations

Our study has several limitations. Firstly, it was conducted as a single-center, hospital-based study, which may have resulted in the underrepresentation of milder cases typically managed in outpatient settings, as these cases were not included in our cohort. Additionally, this study’s limited time frame precluded longitudinal follow-up of all patients, preventing us from capturing potential disease progression or treatment outcomes. Furthermore, we lacked complete data on anti-DSG1 and anti-DSG3 antibodies, as well as DIF results for all patients. However, these data were missing in only a small subset of patients, thus allowing us to draw meaningful conclusions. Nonetheless, our findings regarding antibody titers and DIF studies should be interpreted cautiously, as they cannot be considered fully conclusive. Lastly, we could not assess the evolution of antibody titers throughout the disease and during treatment, further limiting our understanding of their role in disease progression.

## 5. Conclusions

Pemphigus is associated with numerous comorbidities, which are observed both before diagnosis and during the treatment. Developing or aggravating the state of cardiovascular and metabolic diseases, especially in patients treated with systemic GCSs, could be reduced by relatively standard procedures, such as regular blood pressure, glucose, and lipid levels measurements. It is mandatory to provide patients with a multidisciplinary approach. Accurate analysis of the clinical condition of the patients, as well as the results of the ELISA panel or DIF, is crucial for a successful diagnostic and therapeutic process.

## Figures and Tables

**Table 1:** Comorbidities in patients with PV and PF described in the medical history during the diagnostic process.

Comorbidity	Pemphigus Vulgaris (N/%)	Pemphigus Foliaceus (N/%)
Arterial hypertension	51/41	9/50
Hypercholesterolemia	14/27	3/17
Diabetes mellitus	10/20	2/11
Coronary artery disease and/or cardiac failure (including cardiac infarction)	7/14	3/17
Thrombosis and/or pulmonary thromboembolism	4/8	0/0
Obesity	4/8	0/0
Cerebral vascular accident	2/4	0/0
Atherosclerosis	1/2	0/0
Osteoporosis	6/12	1/6
Hypothyroidism	4/8	2/11
Hyperthyroidism	3/6	1/6
Depression	2/4	1/6
Dementia	0/0	1/6
Alcohol dependence syndrome	1/2	0/0
Chronic kidney disease	2/4	0/0
Gout	2/4	0/0
Nephrolithiasis	1/2	0/0
Cholecystectomy	5/10	2/11
Colon polyps	2/4	0/0
Inflammatory bowel disease	1/2	1/6
Peptic ulcer disease	1/2	1/6
Drug-induced liver injury	1/2	1/6
Bronchial asthma	2/4	2/11
Emphysema	1/2	0/0
Uterine fibroid	1/2	0/0
Psoriasis	0/0	1/6
Vulvar lichen sclerosus	1/2	0/0
Cataract	0/0	1/6

**Table 2:** Neoplasms in patients with PV and PF described in the medical history during the diagnostic process.

Neoplasm	Pemphigus Vulgaris (N/%)	Pemphigus Foliaceus (N/%)
Any neoplasm in the medical history	7/14	3/17
Thyroid cancer	2/4	0/0
Breast cancer	1/2	1/6
Melanoma	1/2	0/0
Polycythemia vera	1/2	0/0
Multiple myeloma	1/2	0/0
Colorectal cancer	1/2	0/0
Endometrial cancer	1/2	0/0
Basal cell carcinoma	0/0	1/6
Borderline ovarian tumor	0/0	1/6

**Table 3:** The analysis of ELISA results in patients with pemphigus.

		N	Mean Value	Median Value	SD	*p*
anti-DSG1	PV	29	3.29	2.54	2.09	0.0031
	PF	17	5.37	6.01	1.95	
anti-DSG3	PV	48	5.17	5.27	2.08	-

**Table 4:** Comparison of the correlation between the level of anti-DSG1 and anti-DSG3 antibodies and the age at diagnosis in the study group.

	N	R	*p*
anti-DSG1 vs. age at diagnosis	46	0.32	0.0298
anti-DSG3 vs. age at diagnosis	49	0.06	0.6943

**Table 5:** The analysis of the deposits detected in DIF in patients with PV and PF.

		PV (N/%)	PF (N/%)	*p*
IgG	present	12/24.5	10/55.5	0.0217
	not present	37/75.5	8/44.5	
IgG1	present	17/34.5	10/55.5	0.1627
	not present	32/65	8/44	
IgG4	present	45/92	18/100	0.5673
	not present	4/8	0/0	
C3	present	33/67	11/61	0.7727
	not present	16/33	7/39	
IgA	present	1/2	1/5.5	0.4681
	not present	48/98	17/94.5	

**Table 6:** The analysis of concurrent presence of deposits in DIF and antibodies to DSG1 and/or DSG3 in ELISA.

	Anti-DSG1	Anti-DSG3	Both	*p*
IgG	10	4	8	0.0469
IgG1	9	7	10	1
IgG4	17	16	28	0.0998
C3	11	9	23	0.1342
IgA	1	1	0	0.5959

**Table 7:** The analysis of combinations of deposits in DIF in PV and PF patients.

Combination of Antibodies in DIF	PV (N/%)	PF (N/%)	*p*
IgG + IgG1 + IgG4 + C3	5/10	7/39	0.0054
IgG1 + IgG4 + C3	7/14	2/11	0.7466
IgG4 + C3	16/31	1/6	0.0339

**Table 8:** Treatment of patients with pemphigus in the Department of Dermatology.

Treatment	PV (N/%)	PF (N/%)
Systemic GCSs	46/90	17/94
Topical GCSs	32/63	15/83
Topical calcineurin inhibitor	4/8	3/17
DP	23/45	7/39
RTX	8/16	4/22
Intravenous immunoglobulins	4/8	0/0
Cyclophosphamide	3/6	0/0

## Data Availability

All data are available upon request.
